# Absorption, distribution, metabolism, and excretion of [^14^C]-labeled naloxegol in healthy subjects

**DOI:** 10.5414/CP202276

**Published:** 2015-09-02

**Authors:** Khanh Bui, Fahua She, Michael Hutchison, Åsa Brunnström, Mark Sostek

**Affiliations:** 1 AstraZeneca Pharmaceuticals, Wilmington, DE, USA,; 2 AstraZeneca Pharmaceuticals, Gaithersburg, MD, USA,; 3 AstraZeneca UK Ltd., Cheshire, UK, and; 4 AstraZeneca Pharmaceuticals, Södertälje, Sweden

**Keywords:** constipation, opioid, naloxegol, opioid receptor antagonist

## Abstract

Objective: To characterize the absorption, distribution, metabolism, and excretion of naloxegol, a PEGylated derivative of the µ-opioid antagonist naloxone, in healthy male subjects. Materials and methods: [^14^C]-Labeled naloxegol (27 mg, 3.43 MBq) was administered as an oral solution to 6 fasted subjects. Blood, fecal, and urine samples were collected predose and at various intervals postdose. Naloxegol and its metabolites were quantified or identified by liquid chromatography with radiometric or mass spectrometric detection. Pharmacokinetic parameters were calculated for each subject, and metabolite identification was performed by liquid chromatography with parallel radioactivity measurement and mass spectrometry. Results: Naloxegol was rapidly absorbed, with a maximum plasma concentration (geometric mean) of 51 ng/mL reached before 2 hours after dosing. A second peak in the observed naloxegol and [^14^C] plasma concentration-time profiles was observed at ~ 3 hours and was likely due to enterohepatic recycling of parent naloxegol. Distribution to red blood cells was negligible. Metabolism of [^14^C]-naloxegol was rapid and extensive and occurred via demethylation and oxidation, dealkylation, and shortening of the polyethylene glycol chain. Mean cumulative recovery of radioactivity was 84.2% of the total dose, with ~ 68.9% recovered within 96 hours of dosing. Fecal excretion was the predominant route of elimination, with mean recoveries of total radioactivity in feces and urine of 67.7% and 16.0%, respectively. Unchanged naloxegol accounted for ~ 1/4 of the radioactivity recovered in feces. Conclusions: Naloxegol was rapidly absorbed and cleared via metabolism, with predominantly fecal excretion of parent and metabolites.

ClinicalTrials.gov identifier: NCT01348724 

## Introduction 

Opioid-induced constipation (OIC) is common in patients treated with opioids for moderate or severe pain [1]. The estimated prevalence of OIC ranges between 15 and 90% in patients receiving opioids for noncancer pain [2]. Most patients regard OIC as the most bothersome effect of opioid pain medications and report at least a moderate negative impact on quality of life [3]. 

Opioid-induced constipation is caused by multiple µ-opioid receptor agonist effects on the gastrointestinal (GI) tract [2, 4, 5]. These effects include reduced and impaired GI motility and mucosal secretion, as well as increased absorption of fluid from the gut lumen resulting from the delay in transit time [2, 4, 5]. Additional opioid-mediated actions of clinical relevance include nonperistaltic contractions of the esophagus [6, 7], increases in pyloric [6] and anal sphincter tone [5, 6], sphincter of Oddi constriction [5, 6], and decreased pancreatic and biliary secretions [5, 6]. Collectively, these inhibitory actions of opioid agonists on GI function result in OIC and may also contribute to more serious GI dysfunction, including gastroparesis and ileus [6], fecal impaction [5], and intestinal pseudoobstruction [5, 6]. 

Standard treatment with laxatives or nonpharmacologic interventions (such as increased dietary fiber and fluid intake) does not provide adequate symptom relief for many patients [8, 9]. Conventional laxatives also do not fully address the range of underlying agonist effects of opioids on the GI tract or symptoms associated with delayed gastric emptying, such as bloating, nausea, and satiety; moreover, some chronic laxative use may be associated with electrolyte imbalance and abdominal cramp-like pain [8, 10]. 

Naloxegol (previously known as NKTR-118) is a polyethylene glycol conjugated (PEGylated) derivative of naloxone that has been approved as an oral, once-daily treatment for constipation associated with the administration of opioid analgesics [11]. PEGylation reduces the passive permeability of naloxegol and renders the compound a substrate for the P-glycoprotein transporter [12, 13], thus restricting its access to the brain by PGP-mediated efflux at the blood-brain barrier but maintaining its peripheral opioid receptor antagonist properties. Because the effects of naloxegol are limited to the periphery, the central analgesic properties of opioid agonists are maintained [14]. Naloxegol inhibits the µ-opioid receptor with nM potency [15], is metabolized by CYP3A4 [16], and is a pharmacokinetically relevant substrate only for the PGP transporter [12]. Data from in-vivo metabolism studies demonstrated that all human metabolites were present in mouse, rat, and dog [12]. 

The clinical efficacy of naloxegol was established in two identical randomized, double-blind phase 3 studies conducted in patients with OIC taking opioids for noncancer pain [17]. A significant increase in responder rates occurred with naloxegol 25 mg once daily compared with placebo (naloxegol, 40 – 44%; placebo, 29%) [17], as well as in subsets of patients with inadequate response to conventional laxatives (naloxegol, 47 – 49%; placebo, 29 – 31%) [17]. Naloxegol did not affect opioid-mediated analgesia or daily opioid use, with few instances of investigator-reported opioid withdrawal syndrome [17]. 

**Figure 1.**
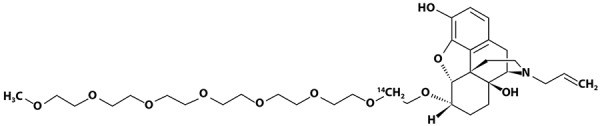
Structure of [^14^C]naloxegol.

The objective of this study was to assess the absorption, distribution, metabolism, and excretion of orally administered [^14^C]-labeled naloxegol (Figure 1) in healthy male volunteers. 

## Materials and methods 

### Radiolabeled naloxegol 

[^14^C]-Naloxegol (Figure 1) was prepared as an oral solution (0.1 M citrate buffer, 10 mL) at a nominal total dose of 25 mg (3.20 MBq ± 10% (78 – 95 µCi ± 10%)). Upon release, the material showed 108% of label claim (27 mg, 3.43 MBq), which was within the acceptance criterion. The actual dose of naloxegol administered was 27 mg. Bioequivalence between the solution and phase 3 tablet formulations was demonstrated previously [18]. 

### Subjects 

Eligible participants were healthy men 50 – 65 years of age. Women were not included because sex differences in the PK of naloxegol were not observed in other clinical studies [14, 19]. 

Inclusion criteria were regular daily bowel movements (self reported; ≥ 1 stool per day), clinically normal physical and laboratory findings, calculated creatinine clearance > 60 mL/min, body mass index (BMI) 18 – 30 kg/m^2^, and body weight ≥ 50 kg. Exclusion criteria were ongoing GI pathology or a history of GI surgery, bowel perforation, fecal incontinence, GI obstruction, chronic idiopathic constipation, clinically important diverticular disease, or any other active disorder associated with chronic diarrhea or intermittent loose stools or constipation; known or suspected history of drug or alcohol abuse; participation in any radiolabeled study within 5 years, radioactivity monitoring as part of profession, or exposure to radiation levels > 5 mSv in the last year, > 10 mSv over the last 5 years, or a total of > 1 mSv per year of life. Current smokers and those who had smoked or used nicotine products within 3 months before screening were excluded, as were individuals with an excessive intake of caffeine-containing drinks (e.g., coffee, tea, caffeine-containing energy drinks, and cola), defined as > 5 cups of coffee or equivalent per day. 

Use of medications or herbal products with enzyme-inducing properties was prohibited within 3 weeks prior to [^14^C]-naloxegol dosing. Use of any medication other than acetaminophen was prohibited within 2 weeks of dosing. Use of products containing grapefruit or Seville oranges was prohibited within 1 week of dosing. 

### Study design and assessments 

This was a phase 1, open-label, single-arm, noncomparative, single-dose, single-center, mass-balance study. The study was conducted at Quintiles Drug Research Unit, Guy’s Hospital, London, United Kingdom from June 21, 2011 to September 7, 2011. The study protocol, amendments, and informed consent forms were reviewed and approved by an independent ethics committee. The study was conducted in accordance with the Declaration of Helsinki, and all subjects provided written informed consent before study participation. 

The study consisted of a screening visit, a residential visit, and a follow-up visit. During the screening visit, subjects provided informed consent and medical/surgical history; underwent physical examination and measurement of vital signs, serology, hematology, clinical chemistry, urinalysis, and 12-lead electrocardiogram (ECG); and underwent screening tests for drugs of abuse, alcohol, and cotinine. Subjects also completed a Columbia-Suicide Severity Rating Scale (C-SSRS) assessment [20], an instrument routinely used for drugs with central nervous system effects. 

Following the screening visit, each subject was admitted to the study center on day –1 for an 11-day residential period. On the morning of day 1, subjects received a single oral dose of [^14^C]-naloxegol 27 mg (3.43 MBq) after a 10-hour fast. The dose was administered as a 10-mL oral solution. The container was rinsed repeatedly with water, and subjects drank the rinses, with the total volume not exceeding 240 mL. Blood sampling for pharmacokinetic (PK) analysis was performed predose from day 1 at 0.25, 0.5, 1, 1.5, 2, 3, 4, 6, 8, 10, 12, 16, 24, 36, 48, 72, 96, 120, 144, 168, 192, 216, and 240 hours postdose. Blood sampling for metabolite identification was performed predose on day 1 and at 0.5, 2, 4, 6, 12, and 24 hours postdose. Fecal collection occurred predose from –24 to 0 hours, postdose from 0 to 24 hours, and daily thereafter. Urine collection occurred from –12 to 0 hours predose, at 0 to 6, 6 to 12, and 12 to 24 hours postdose, and daily thereafter to ensure high recovery of radioactive material. Vital sign examinations and ECG were performed on day –1, predose on day 1, and 1, 2, and 4 hours postdose. The C-SSRS was administered on day –1 and 48 hours postdose, and laboratory tests were performed on day –1 and 48 hours postdose. 

A follow-up visit was conducted 5 – 7 days after collection of the last urine and/or fecal sample and involved physical examination, vital signs, ECG, laboratory testing, and C-SSRS. AEs were recorded from the time of informed consent throughout the study. 

### Determination of naloxegol concentrations in plasma and urine 

Concentrations of naloxegol were determined by validated bioanalytical methods using liquid chromatography (LC; Shimadzu LC20AD LC system, Shimadzu Scientific Instruments, Columbia, MD, USA) followed by tandem mass spectrometric detection (LC-MS/MS; API 5000, AB SCIEX, Framingham, MA, USA). Plasma samples (0.1 mL per determination) underwent solid-phase extraction and were separated by liquid chromatography using a C18 column (50 × 3 mm, 3 µm particle size). Calibration curves extended over the concentration range of 0.1 – 50 ng/mL, with a lower limit of quantification of 0.1 ng/mL. Urine samples (0.05 mL per determination) were treated with Triton X-100 (The Dow Chemical Company, Midland, MI, USA) and assayed by direct dilution and LC-MS/MS using the same chromatographic conditions as for plasma. Calibration curves were validated over the concentration ranges of 25 – 5,000 ng/mL, with a lower limit of quantification of 25 ng/mL. Both methods used stable labeled [^13^C_6_]-naloxegol as the internal standard. 

### Metabolite identification and quantification 

Metabolite profiles were monitored in plasma (0.5, 2, 4, and 6-hour samples), pooled urine (0 – 24 hours), and pooled fecal homogenates (0 – 120 hours). Samples were separated by liquid chromatography using a C18 column (3.5-µm particle size) at a flow rate of 1.0 mL/min. Column effluent was split 1 : 20 to the radioactivity detector (95%) and mass spectrometer (5%). Scintillation analyses were performed on a Tri-carb 1900TR liquid scintillation analyzer (Perkin Elmer, Boston, MA, USA). Radioactivity was measured for 3 minutes and reported as disintegrations per minute. Mass spectrometry was performed on an LTQ linear ion trap (Thermo Electron Corporation, San Jose, CA, USA). Xcalibur 2.0 SR2 (Thermo Electron Corporation) software was used for data acquisition, processing, and control of the mass spectrometer. Radiochromatographic peaks representing a fraction of dose < 1% were not structurally characterized unless the mass spectral interpretation was obvious. Characterized peaks were assigned a metabolite number based on the metabolite characterization data across a number of species; uncharacterized peaks were assigned as MXn. 

### Statistical analysis 

The PK analysis set included all subjects who received [^14^C]-naloxegol and had ≥ 1 postdose PK measurement. PK parameters, including half-life (t_1/2_), maximum concentration (C_max_), time to maximum concentration (t_max_), area under the plasma concentration-time curve (AUC), apparent oral clearance (CL/F), and apparent volume of distribution (Vz/F), were calculated by Quintiles (Overland Park, KS, USA) using noncompartmental PK methods and WinNonlin software (Pharsight Corporation, Mountain View, CA, USA). Total [^14^C] radioactivity concentrations in plasma and whole blood, the amount and the fraction of [^14^C] radioactivity excreted in urine and feces, and the amount and fraction of naloxegol in urine were also calculated. Ratios of plasma naloxegol to plasma [^14^C] radioactivity and of whole blood radioactivity to plasma [^14^C] radioactivity were calculated, and distribution into red blood cells was assessed. The fraction of administered dose, characterized as a metabolite in urine and feces, was obtained by multiplying the excreted fraction of dosed radioactivity recovered in the excreta samples, with the fraction of drug-related material detected as parent compound or metabolite by radioactivity monitoring. PK and cumulative urine and fecal recovery variables were summarized using descriptive statistics. 

The safety-analysis set included all patients who received [^14^C]-naloxegol and had any postdose data available. Safety variables were analyzed using descriptive statistics. 

No formal power calculation was performed to estimate the sample size; a sample size of 6 subjects is typical in mass-balance studies. 

## Results 

### Subjects 

The study consisted of 6 male volunteers; 5 were white and 1 was black. Mean age was 56 years (range 50 – 63), and mean BMI was 24.6 kg/m^2^ (range 20.6 – 26.7). 

### Absorption 

**Figure 2.**
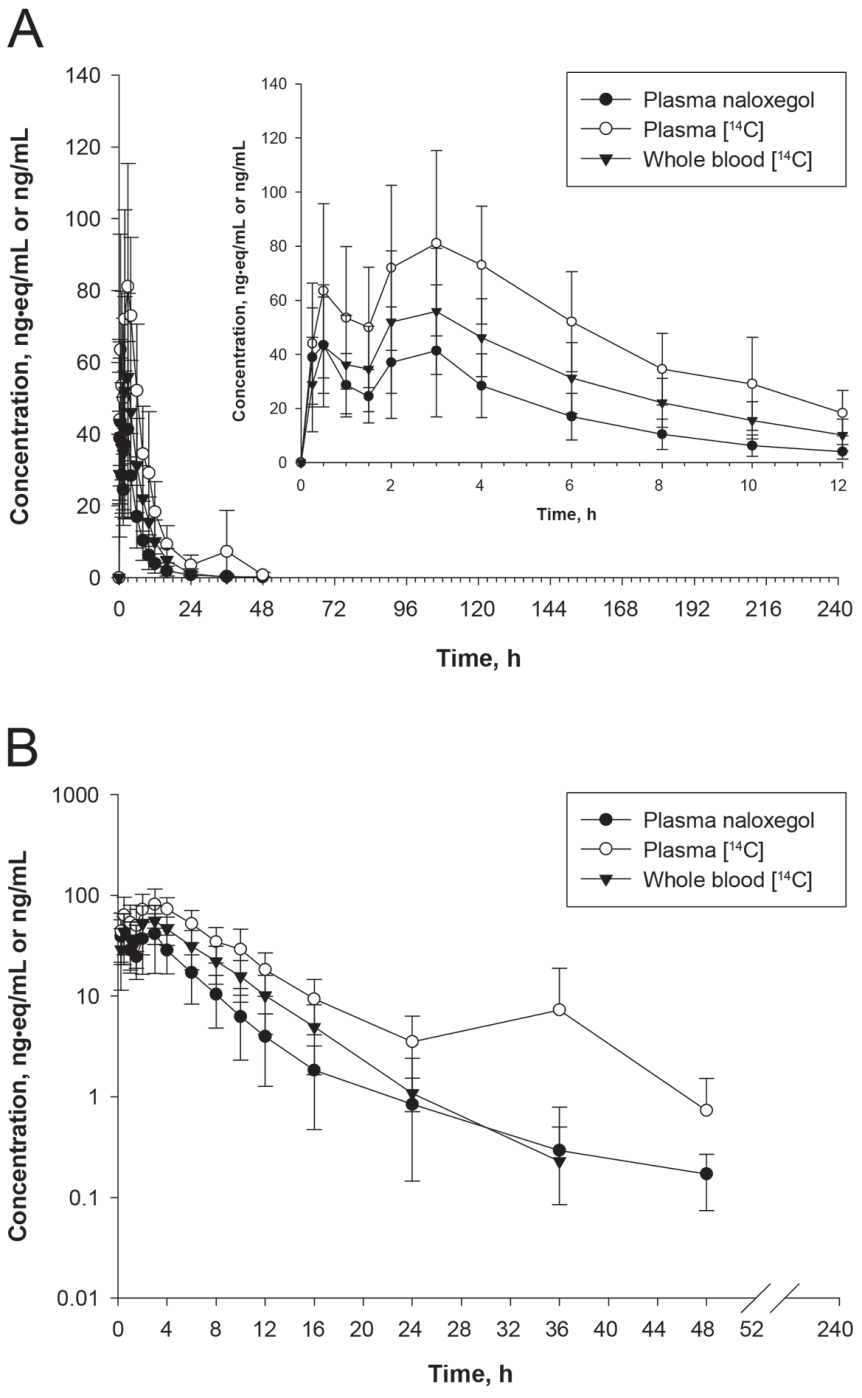
Concentration-time plots for naloxegol on (A) linear and (B) semilogarithmic scales. Values represent the mean ± standard deviation of 6 subjects. Mean concentrations below the lower limit of quantitation are not presented.

Following administration of [^14^C]-naloxegol, naloxegol concentrations and radioactivity were quantifiable in plasma and whole blood in all subjects at the first postdose time point (0.25 hours), indicating rapid absorption (Figure 2). In most subjects, plasma concentrations remained quantifiable for up to 48 hours postdose. Mean naloxegol C_max_ in plasma was 51 ng/mL, with a median t_max_ of 1.74 hours (range, 0.25 – 3.02) (Table 1). 

In several subjects, multiple peaks were observed in both the naloxegol and plasma [^14^C] concentration-time profiles after naloxegol administration. The first appeared at ~ 0.25 – 0.5 hours and the other appeared at ~ 4 hours. The secondary peak in the observed naloxegol and [^14^C] plasma concentration-time profiles is likely due to enterohepatic recycling of parent naloxegol. 

The fraction of dose absorbed was estimated as the sum of the 15.4% of the dose excreted in urine and the 44.2% of the dose recovered in the feces and attributable to identified metabolites, giving a value of ~ 60% (Table 2). 

### Distribution to red blood cells 

The mean ratio of whole blood radioactivity to plasma radioactivity ranged from 0.563 to 0.703 over the first 10 hours after dosing and was relatively independent of time. Because plasma represents ~ 55% to 60% of whole blood, these ratios suggest that very little of the radioactivity in the vascular compartment is associated with red blood cells. This is confirmed by the total radioactivity associated with red blood cells, which ranged from 0 to 13.8%, indicating that naloxegol and metabolites do not distribute to red blood cells to any significant extent. 

### Metabolism 

Mean plasma radioactivity concentrations exceeded mean naloxegol plasma concentrations at all time points, indicating the presence of metabolites in the circulation. The proportion of plasma radioactivity attributable to naloxegol (based on concentrations of naloxegol measured by LC-MS/MS and total radioactivity) was time dependent and decreased from ~ 90% at 0.25 hours to 70% at 0.5 hours and 9% at 16 hours, indicating rapid and sustained presence of metabolites in the circulating system. Plasma naloxegol C_max_ and AUC values accounted for 60.2% and 32.8%, respectively, of the equivalent values calculated for plasma radioactivity. Mean t_1/2_ was similar between plasma naloxegol and plasma radioactivity (7.88 and 7.28 hours). The exposure ratios presented represent minimum values, because no correction for [^14^C]-naloxegol content was applied in the quantitation of naloxegol by LC-MS/MS. Hence, total concentrations were slightly underestimated, resulting in an error of < 5%. 

### Metabolite characterization and quantification 

**Figure 3.**
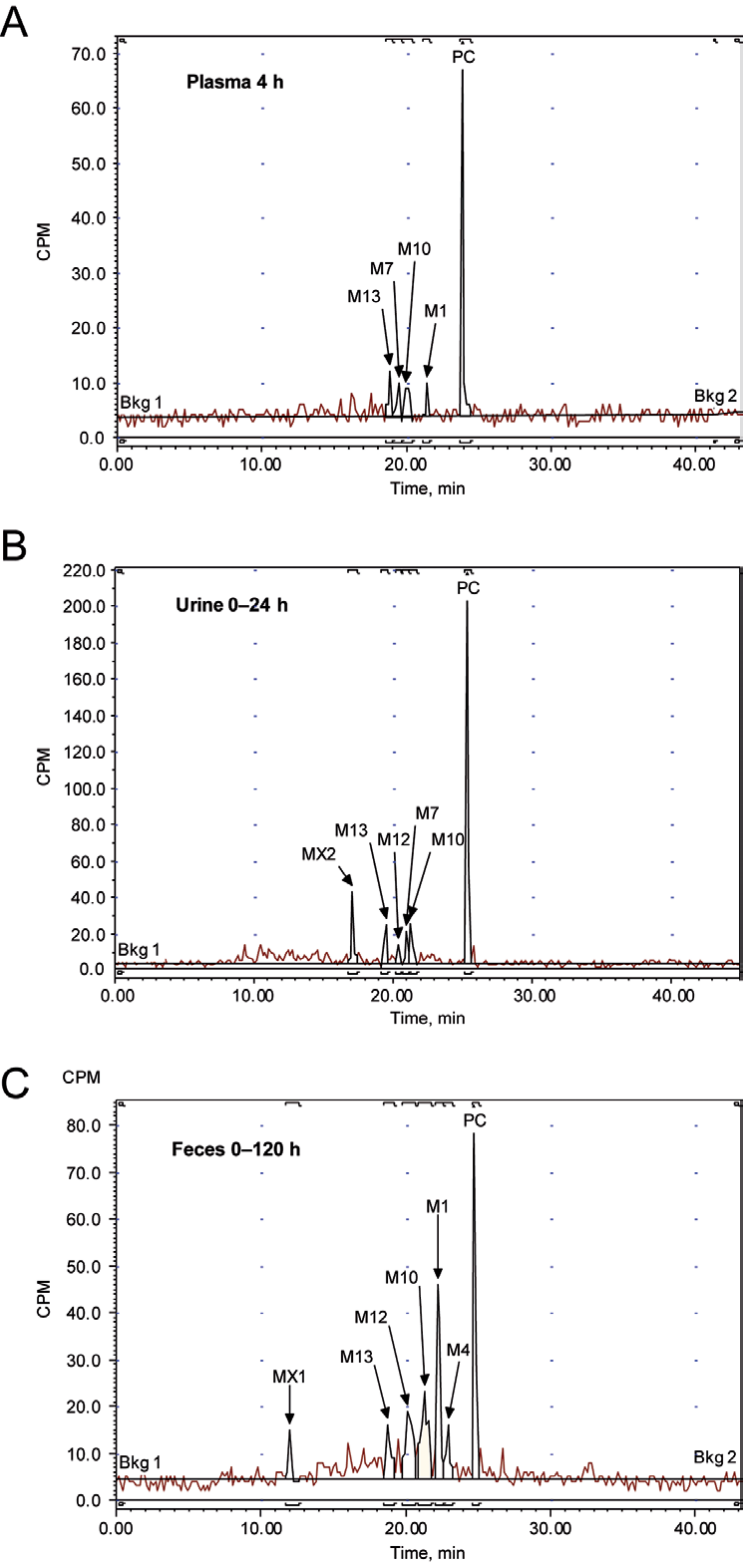
Radiochromatograms from analysis of metabolites in pooled (A) plasma, (B) urine, or (C) feces. Bkg = background; CPM = counts per minute; PC = parent compound.

**Figure 4.**
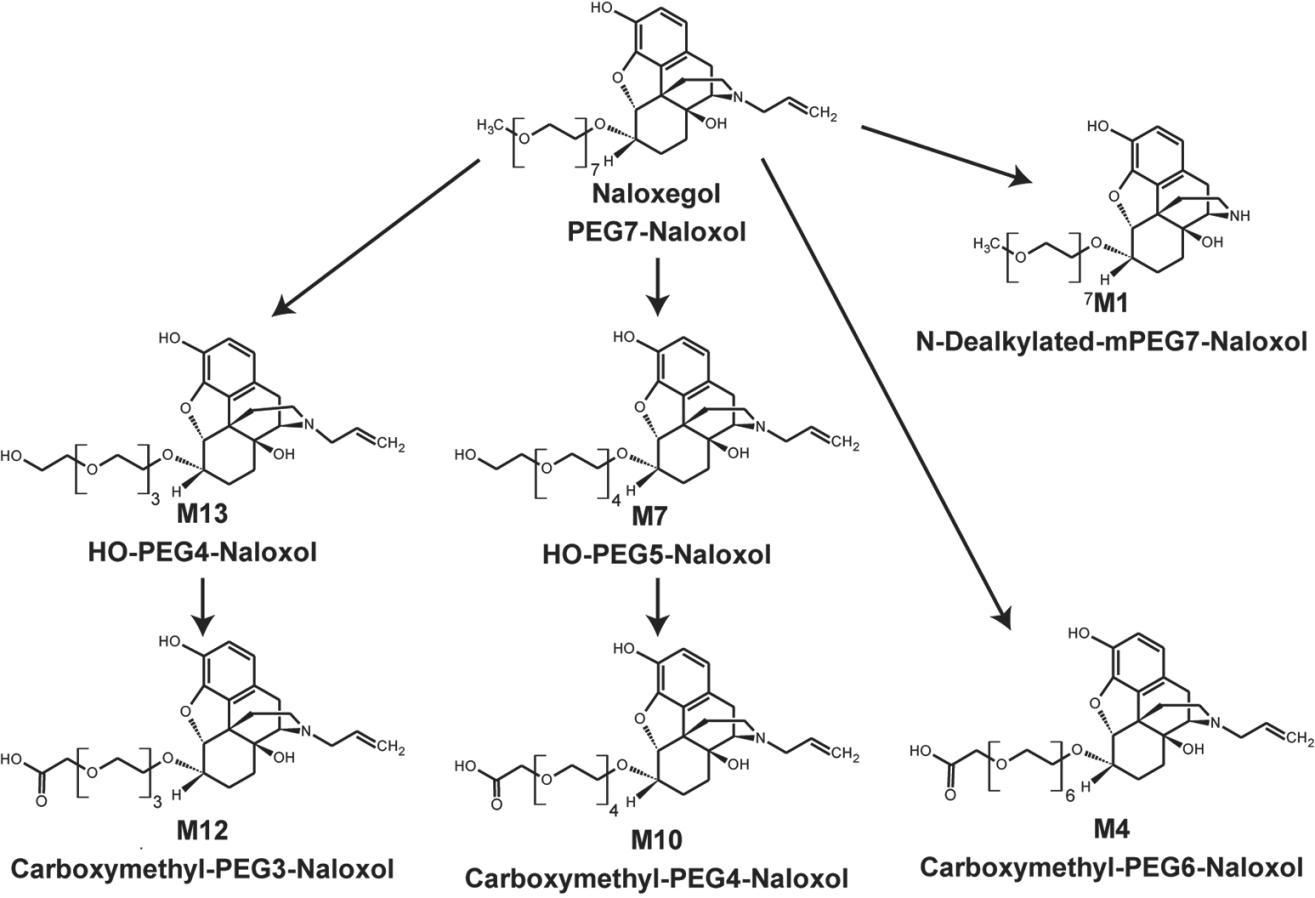
Structures of the human metabolites of naloxegol.

Six metabolites were characterized in urine, feces, and plasma (M13, M12, M7, M10, M1, and M4) (Figure 3, Figure 4) (Table 2). The major metabolic pathways accounted for metabolism of > 32% of the administered dose. These included formation of M13 and M7 from partial losses of the PEG chain, M4 by demethylation and oxidation to form a carboxylic acid at the end of the PEG chain, M12 and M10 by PEG chain shortening combined with oxidation, and M1 by N-dealkylation. Two uncharacterized peaks (MX1 and MX2) together represented 4% of the dose. None of the metabolites identified had the PEG chain completely cleaved. 

Circulating metabolites accounted for 25% of the plasma drug-related material 2 hours after dosing and 36% of the material 4 hours after dosing (Table 3). The M10 metabolite was the most abundant in plasma (9.5% of total plasma drug-related material at 2 hours and 12% at 4 hours), followed by M13 (2 hours, 4.7%; 4 hours, 10%) and M7 (2 hours, 6.3%; 4 hours, 8.2%). At other times radioactivity levels were so low that naloxegol was the only identifiable component. 

### Excretion (mass balance) 

**Figure 5.**
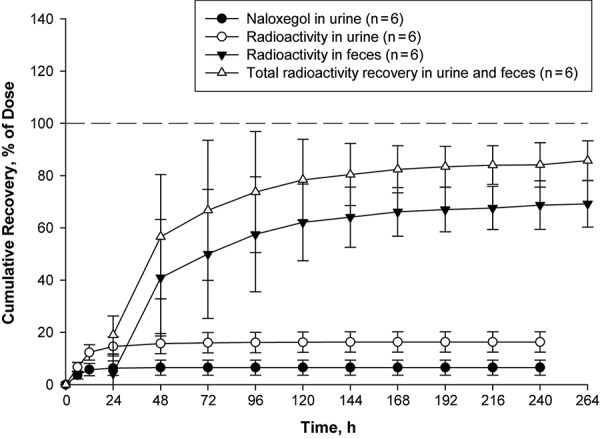
Arithmetic mean recovery of naloxegol and radioactivity over time. Error bars represent standard deviation.

The arithmetic mean recoveries for naloxegol in urine and radioactivity in urine and feces are presented in Figure 5. The geometric mean of cumulative radioactivity recovery in urine and feces combined was 84.2% of dose, and the major fraction of radioactivity was recovered within 96 hours of dosing. The geometric mean recovery of total radioactivity in feces and urine was 67.7% and 16.0%, respectively, indicating that fecal elimination is the primary elimination pathway. Over 240 hours, the average (geometric mean) of recovered unchanged naloxegol dose in urine (as measured by LC-MS/MS) was 5.9%, indicating that urinary clearance is a minor pathway for the elimination of parent drug. Overall ~ 1/4 of the radioactivity in feces was excreted as the parent compound. 

### Safety 

Adverse events occurred in 2 subjects and consisted of mild diarrhea (n = 2) and mild fatigue (n = 1). All AEs resolved without intervention. There were no AEs leading to discontinuation, no serious AEs, and no deaths. No clinically relevant changes in vital signs, ECG, or physical examination were observed in any subject, and no subject reported suicidal behavior or ideation as determined by the C-SSRS. 

## Discussion 

Naloxegol administered as an oral solution was rapidly absorbed, and clearance was predominantly metabolic. The major metabolic pathways of naloxegol involved shortening of the PEG chain and/or oxidation of the PEG moiety. The other important route was N-dealkylation. These transformations together accounted for the majority of the total excreted radioactivity (Table 2). In preclinical safety studies conducted in rats and dogs, pooled samples from the no-effect dose levels at steady-state were compared with pooled human samples on day 8 of naloxegol dosing (25 mg). The 4 metabolites found in human plasma (Table 3) were present at much higher concentrations in animals than in patients, with exposure ratios of 6- to 205-fold [21]. 

Cleavage of the PEG chain yielded metabolites containing 3 – 6 PEG monomers; all circulating metabolites had > 3 PEG repeats. Previous data suggest that PEG chains with 3 subunits are sufficient to reduce penetration to the central nervous system [22]. No metabolite constituted > 14% of the total dose. Approximately 16% of the naloxegol dose was excreted unchanged in feces, which may include unabsorbed material. 

Fecal elimination was the major pathway of elimination for drug-related material; renal elimination constituted a minor pathway. Although the metabolic profile of orally administered naloxone has not been fully characterized in humans, it shares similarities with naloxegol. Radiolabeled naloxone (7, 8-[^3^H]) at an oral dose of 0.1 mg was rapidly absorbed, with maximum plasma radioactivity at 30 minutes [23]. Excretion was also rapid, with 24 – 37% of the dose appearing in urine by 6 hours [23]. Metabolism by N-dealkylation and 6-keto reduction was suggested by detection of the parent compound naloxone, as well as the metabolites 7,8-dihydro-14-hydronormorphinone and N-allyl-7,8-dihydro-14-hydronormorphinone, in urine [24, 25]. N-dealkylation was also observed for naloxegol (Figure 4). 

Naloxegol was generally well tolerated in these healthy male subjects, and there were no reported safety concerns. Few treatment-emergent AEs were observed in this study. A potential limitation of the study was the discrepancy in the recovery of naloxegol in urine when naloxegol was measured by LC-MS/MS vs. radioprofiling, which may be attributable to the potential for lower sensitivity with radiochromatography. 

## Conclusion 

In summary, naloxegol was rapidly absorbed and cleared mainly by metabolism in healthy male volunteers in this phase 1 trial. Naloxegol and its metabolites were excreted primarily through the fecal route. The human metabolites identified have been shown to be present in the species selected for the toxicological evaluation of naloxegol. 

## Acknowledgments 

This study was supported by AstraZeneca LP (Wilmington, DE). Editorial support was provided by Valerie P. Zediak, PhD, and Judy Fallon, PharmD, CMPP, from Complete Healthcare Communications, Inc. (Chadds Ford, PA), and funded by AstraZeneca LP. 

## Conflict of interest 

F. She, M. Hutchison, and M. Sostek are employees of AstraZeneca. K. Bui was an employee of AstraZeneca at the time this work was performed, and is currently employed as a contractor for AstraZeneca. K. Bui, M. Hutchison, and M. Sostek are shareholders of AstraZeneca. Å. Brunnström was an employee of AstraZeneca at the time this work was performed, and is currently an employee of Swedish Orphan Biovitrum AB, Stockholm, Sweden. 

**Table 1.** PK parameters of naloxegol and radioactivity in plasma and whole blood (n = 6).

Parameter^a^	Plasma naloxegol	Plasma radioactivity	Whole blood radioactivity
AUC_0–_∞, ng×h/mL	233 (61.9)	710 (49.2)	392 (44.2)
C_max_, ng/mL	51.1 (38.3)	84.8 (40.3)	57.5 (40.8)
t_max_, h^b^	1.74 (0.25 – 3.02)	2.23 (0.50 – 4.02)	2.20 (0.50 – 4.02)
t_1/2_, h	7.88 (46.4)	7.28 (41.4)	3.66 (27.5)
λ_z_, h^–1^	0.0880 (46.5)	0.0952 (41.4)	0.190 (27.5)
Vz/F, L	1,320 (54.6)	399 (25.2)	364 (22.6)
CL/F, L/h	116 (61.9)	38.0 (49.3)	69.0 (44.3)

λ_z_ = terminal elimination rate constant; AUC_0–_∞ = area under the curve from time zero extrapolated to infinity; CL/F = apparent oral clearance; C_max_ = maximum concentration; PK = pharmacokinetic; t_max_ = time to maximum plasma concentration; t_1/2_ = apparent elimination half-life; Vz/F = apparent volume of distribution. ^a^Values are geometric mean (geometric coefficient of variation in percent) unless otherwise noted. ^b^Values are median (min, max).

**Table 2.** Metabolite identity and excretion.

Metabolite	Proposed transformation	Site of detection	Fraction of dose in feces 0 - 120 h, %	Fraction of dose in urine 0 - 24 h, %
Naloxegol	Unchanged	P, F, U	16.2	9.9
MX1	Not available	F	2.2	–
MX2	Not available	U	–	1.8
M13	Partial loss of PEG	P, F, U	4.5	1.1
M12	Partial loss of PEG and oxidation	F, U	9.1	0.4
M7	Partial loss of PEG	P, U	–	0.7
M10	Partial loss of PEG and oxidation	P, F, U	10.9	1.5
M1	Dealkylation	P, F	13.7	–
M4	Partial loss of PEG and oxidation	F	3.8	–
Fraction of dose			60.4	15.4

F = feces; MX = uncharacterized metabolite; P = plasma; PEG = polyethylene glycol; U = urine.

**Table 3.** Summary of metabolite profile data in plasma from healthy male volunteers after oral administration of 27 mg (3.43 MBq) naloxegol.

Metabolite		Fraction of total peak area in plasma, %^a^				
	R_t_^b^	R_t_, min	0.5 hours^c^	2 hours	4 hours	6 hours
Naloxegol	1.0	23.9	100	75	64	100
M13	0.79	19.0	ND	4.7	10	ND
M7	0.82	19.6	ND	6.3	8.2	ND
M10	0.84	20.2	ND	9.5	12	ND
M1	0.90	21.5	ND	4.8	5.5	ND

ND = not detected (signal-to-noise ratio < 3 in the radiochromatogram); R_t_ = retention time. ^a^Percentage of the total peak area in the radiochromatograms. ^b^Relative to parent, calculated as R_t_ metabolite/R_t_ naloxegol. ^c^Collection time after dosing.
